# Role of human metapneumovirus glycoprotein G in modulation of immune responses

**DOI:** 10.3389/fimmu.2022.962925

**Published:** 2022-07-25

**Authors:** Thangam Sudha Velayutham, Teodora Ivanciuc, Roberto P. Garofalo, Antonella Casola

**Affiliations:** ^1^ Department of Pediatrics, University of Texas Medical Branch, Galveston, TX, United States; ^2^ Department of Microbiology and Immunology, University of Texas Medical Branch, Galveston, TX, United States; ^3^ Sealy Institute for Vaccine Sciences, University of Texas Medical Branch, Galveston, TX, United States

**Keywords:** RNA virus, hMPV G protein, lung disease, immune response, mouse model

## Abstract

Human metapneumovirus (hMPV) is an important pathogen responsible for acute respiratory tract infections in children, the elderly, and immunocompromised patients, with no effective treatment or vaccine currently available. Knowledge of virus- and host-specific mechanisms contributing to the pathogenesis of hMPV infection is still limited. Studies have shown that hMPV surface glycoprotein G is an important virulence factor, by inhibiting innate immune signaling in airway epithelial cells and immune cells. In this study, we investigated the role of G protein in modulating innate and adaptive immune responses in mice infected with a recombinant virus with deletion of G protein (rhMPV-ΔG). Results show that rhMPV-ΔG was strongly attenuated, as it did not induce significant clinical disease, airway obstruction and airway hyperresponsiveness (AHR), compared to infection with a control strain (rhMPV-WT). By analysis of cells in bronchoalveolar fluid and lung tissue, as well as cytokine production, we found that G protein mediates aspects of both innate and adaptive immune responses, including neutrophils, dendritic cells, natural killer cells and B cells. Lung T cells recruited in response to rhMPV-ΔG had a significantly higher activated phenotype compared to those present after rhMPV-WT infection. Despite highly attenuation characterized by low levels of replication in the lung, rhMPV-ΔG was able to induce neutralizing antibodies and to protect mice from a secondary hMPV challenge. However, challenged mice that had received rhMPV-ΔG as primary infection showed some signs of lung disease at the earliest time points, which were less evident in mice that had received the rhMPV-WT strain as primary infection. These results demonstrate some of the mechanisms by which G protein could contribute to airway disease and modulate immune response to hMPV infection.

## Introduction

Human metapneumovirus (hMPV) is a non-segmented negative sense RNA virus belonging to the *Pneumoviridae* family, according to the new taxonomy of the order *Mononegavirales* (1). It represents one of the leading respiratory viruses responsible for respiratory tract infections in infants, the elderly, individuals with chronic conditions (asthma, cancer, etc.), and immunocompromised patients (2–4). It is a common pathogen identified among children < than 5 years of age than among older children, accounting for nearly 5–15% of all the hospitalizations, outpatient and emergency department visits for lower respiratory tract infections (5–8). Hospitalization rates (~10%) for hMPV among adults aged ≥ 50 years, have been shown to be similar to those of respiratory syncytial virus (RSV) and influenza (9). A study found that during the influenza A H1N1 2009/10 pandemic and early post pandemic phase both hMPV and RSV were leading to a higher clinical disease burden than influenza, prompting for routine testing for hMPV (10). A more recent study covering the 2014-2019 viral seasons has shown that rates of children hospitalization due to hMPV were significantly higher than both RSV and influenza (11). Although few studies on potential vaccine candidates for hMPV have been reported, no vaccine or specific therapy are currently available and our current understanding of the virus- and host-specific mechanisms contributing to the pathogenesis and immune responses to hMPV infection remains limited (3, 8, 12, 13).

hMPV comprises two genetic groups, A and B, with subgroups A1, A2, B1 and B2 (14). The viral genome encodes nine proteins, specifically nucleoprotein (N), phosphoprotein (P), and large RNA-dependent RNA polymerase protein (L), matrix protein (M), M2-1 and M2-2 proteins, and three surface transmembrane proteins including the attachment (G), the small hydrophobic (SH), and the fusion (F) proteins (3). hMPV G protein is a type II mucin-like glycosylated protein (4). The membrane anchor of G protein proximal to the N terminus and its C terminus is oriented externally.

Although the postulated function of hMPV G protein is viral attachment to target cells, hMPV F protein alone is sufficient to mediate attachment and fusion in the absence of other surface proteins including G (15). In addition, the interaction of F with cellular integrin receptors is independent of G protein (16), suggesting that G protein plays a minor role in hMPV attachment. Although lack of G protein does not significantly decrease the ability of hMPV to replicate *in vitro*, as we and others have shown (15, 17), a recombinant virus in which the G protein was deleted (rhMPV-ΔG) exhibited reduced replication in the upper and lower respiratory tract of Syrian hamsters and African green monkeys, making rhMPV-ΔG a suitable vaccine candidate (18, 19). In addition, studies by our group and others have identified hMPV G protein as an important virulence factor, responsible for inhibiting innate immune signaling in airway epithelial cells by targeting RIG-I-dependent gene transcription and MAVS activation, and in monocyte-derived dendritic cells by targeting Toll-like receptor 4 (TLR-4)-dependent signaling and hMPV internalization, therefore affecting CD4^+^ T cell activation (17, 20–23). Nonetheless, our knowledge of the functional role of G protein in modulating hMPV-induced lung disease pathogenesis and immune responses is still limited.

In this study, we investigated the contribution of G protein in hMPV lung pathophysiology, immune responses and protection against re-infection using an experimental mouse model. Results of these studies indicate that a recombinant hMPV strain with G protein deletion (rhMPV-ΔG) was strongly attenuated and did not induce significant clinical disease and airway disfunction, compared to a rhMPV wild type strain (rhMPV-WT). Infection by rhMPV-ΔG was associated with a distinct phenotype of cellular immune response in the lung, characterized by increased recruitment of dendritic cells, natural killer cells and B cells, and activated T cells, compared to infection with rhMPV-WT. rhMPV-WT and rhMPV-ΔG strains induced generation of serum neutralizing antibodies, and infection by both strains was protective against a viral challenge with rhMPV-WT.

## Materials and methods

### Viral preparation and titer

hMPV strain CAN97-83 was used as a backbone to create wild-type hMPV (rhMPV-WT) and hMPV lacking G protein (rhMPV-ΔG) by reverse genetics, as previously described (17). These viruses were propagated in LLC-MK2 cells in MEM (without serum) containing 1.0 μg trypsin/ml, and filtered using Millipore filters (Amicon - Ultra-15 Centrifugal Filter Unit 100K) for mice inoculation. Viral and cellular preparations were routinely tested for mycoplasma contamination by PCR and endotoxin levels by Limulus assay. Viral pools were titered by immunostaining, as previously described (24). For lung viral titration, the lungs were collected on different days post infection (p.i.), processed as previously described (24) and titration was performed by TCID_50_ (25).

### Mice infection protocol

Animal protocol was approved by the Institutional Animal Care and Use Committee of the University of Texas Medical Branch at Galveston. All procedures were carried out in accordance with the recommendations in the Guide for the Care and Use of Laboratory Animals of the National Institutes of Health. Six to eight wk-old female BALB/c mice (Harlan, Houston, Texas) were anesthetized with a combination of xylazine (8mg/kg) and ketamine (70mg/kg) and inoculated intranasally (i.n.) with 5x10^6^ pfu of filtered rhMPV (-WT or -ΔG), in a total volume of 50 to 100 μl. As mock treatment, the mice were inoculated with the same volume of virus-free medium (control/mock-infected mice). For challenge infection, the mice infected with mock, rhMPV-WT and rhMPV-WT, were challenged with 5x10^6^ pfu of rhMPV-WT six weeks after primary infection.

### Clinical Disease

Body weight was measured daily over a period of 14 days p.i. with rhMPV (-WT or -ΔG) and also post challenge infection, as an indicator of disease progression. The severity of illness in mice was also scored daily using a standardized 0-to-5 grading system (26).

### Airway obstruction and hyperresponsiveness (AHR)

Airway obstruction and AHR were assessed in conscious unrestrained mice at various time points (day 3 to 14) p.i. using whole-body plethysmography (Buxco, Troy, NY) to record enhanced ^pause^ (Penh), as previously described (27). The accuracy of the Penh value as an index of airway obstruction has been previously validated in animal models as it correlates well with pulmonary airflow resistance and obstruction (28, 29). It is established as a useful tool for the evaluation of the functional consequences of respiratory viruses on airway functions (30–33). Baseline Penh values with or without infection was used to define airway obstruction while airway resistance in response to methacholine challenge defined AHR. Briefly, each mouse was placed in a plastic chamber and allowed to acclimate to the chamber, and then baseline readings were recorded to determine airway obstruction. Mice were subsequently exposed to aerosolized PBS containing increasing concentrations of methacholine (0, 3.125, 6.25, 12.5, 25, and 50 mg/ml; Sigma, USA) for 2 min and respiratory activity was recorded for another 3 min post nebulization. The highest Penh value, representing airway resistance, obtained during each methacholine challenge was expressed as a proportion of the basal Penh value in response to PBS challenge.

### Bronchoalveolar lavage (BAL) fluid analysis

At different days p.i. (6h, 15h, days 1, 2, 3, 5, 7, 9, and 14) BAL fluid was collected by washing the lungs with 1 ml of chilled PBS. An aliquot of BAL samples was subjected to cytospin, and differential cell count was performed under light microscopy by staining with HEMA3 (Fisher Scientific) and total cell counts were counted with a hemacytometer, and viability was assessed using trypan blue, and rest of the BAL fluid was stored at -80°C without freezing medium additives for further analysis (26, 34). Luminex-based Bio-Plex system (Bio-Rad Laboratories, Hercules, CA) was used to quantify chemokine and cytokine levels in BAL fluid, according to the manufacturer’s instructions. The lower limit of detection for all cytokines measured by this assay is 3 pg/ml. Type I interferons (IFN-α/-β) were quantified using commercial enzyme-linked immunosorbent assays (ELISA), according to the manufacturer’s instructions (PBL, Piscataway, NJ).

### Analysis of lung cellular recruitment by flow cytometry

For flow cytometry analysis, lungs were collected at different time points (days 3, 5, 7 and 9 post-infection) after rhMPV (-WT or -ΔG) or mock infection and digested with collagenase A (0.5 mg/ml PBS; Sigma-Aldrich, St. Louis, MO). Single cell suspension was prepared by passing the cells through nylon mesh, and contaminating erythrocytes were lysed using ACK lysis buffer (Invitrogen). Live gated cells were incubated with Fc block (anti-mouse CD16/CD32) for 30 min to reduce nonspecific binding before addition of Abs (BD Pharmingen, San Diego, CA) against surface markers: anti-CD3ε, -CD4, -CD8β (T cells); -CD19, B220 (B cells); DX5, NK1.1 (NK cells); -CD11c, -CD11b, MHCII, F4/80, Ly6G & Ly6C (Gr-1), and -mPDCA1 (Miltenyi Biotec) (cDCs, pDCs, macrophages, and neutrophils). Relevant isotype control antibodies were used throughout. Samples were stained and run through a FACS Canto flow cytometer (BD Pharmingen, San Diego, CA) and data was analyzed using the FlowJo Software (Tree Star).

### Lung histopathology

Lungs were perfused, fixed in 10% buffered formalin, and embedded in paraffin. Multiple 4 μm-thick sections were stained with hematoxylin & eosin (H&E). Slides were analyzed and scored for alveolitis, interstitial pneumonia and necrosis under light microscopy by two independent investigators [20].

### Lymphocyte activation

Activation of T and B cells were further studied by additional staining with antibodies against -CD44, -CD69, -CD40 and -CD86. Intracellular cytokine staining was performed to detect IFN-γ production by activated CD8+ T cells, as described elsewhere with slight modifications (35). In brief, lung cells were isolated on various days p.i. (days 3, 5, 7 and 9) with rhMPV (-WT or -ΔG) or mock and stimulated with 50 ng/ml PMA and 500 ng/ml ionomycin (Sigma-Aldrich) for 5 h at 37°C. Golgi Stop (BD Biosciences, San Diego, CA) was added during the final 3.5h. The cells were harvested and stained with anti-CD3 and -CD8 antibodies. Cells were fixed, permeabilized and stained with anti-IFN-γ antibodies using the Cytofix/Cytoperm solution (BD Biosciences, San Diego, CA) according to the manufacturer’s instructions. Samples were immediately analyzed by flow cytometry.

### Analysis of lung DC activation markers

A part of the lung single cell suspensions was labeled with CD11c microbeads (Miltenyi Biotec, Auburn, CA) for enrichment of mouse DCs using magnetic activated cell sorting (MACS), and isolated cells were stained with anti-CD11c and anti-MHC-II, in combination with anti-CD40, -CD80, -CD83 and -CD86 (BD-Pharmingen, San Diego, CA). Samples were stained and analyzed by flow cytometry.

### T cell proliferation

For T cell proliferation assays, MACS sorted cDC from rhMPV (-WT or -ΔG) and mock-infected mice (10^5^ cells/well) were cultured for 5 days with CD4+ T cells from DO11.10 mice (2x10^5^ cells/well) in 96-well round bottom microtiter plates. cDC were loaded with 10 μg/mL of OVA peptide (323–339, Genscript, Piscataway, NJ) 2h prior to co-culture with T-cells. The CD4+ T cells were tagged with CFSE prior to culture and after 5 days of co-culture, cells were collected, washed and analyzed by FACS for T-cell proliferation, as determined by increase in CFSE low positive cells (36).

### Determination of anti-hMPV antibodies

Sera samples were collected on weeks 2, 4, 6 p.i. and one week after challenge to determine induction of total IgG and neutralizing antibody response to infection. Total IgG antibody response against hMPV was determined by ELISA. Purified and filtered hMPV strain CAN97-83 was assayed for protein concentration by BCA protein analysis (Pierce Biotechnology, Inc., Rockford, IL), diluted in carbonate-bicarbonate buffer (pH 9.6) to coat 96 well plates (Maxisorb, Nunc, Denmark) with 1 µg/100 µl/well. The plates were incubated at 4°C overnight and ELISA assay was performed as described previously (37) with some modifications. After blocking the plates with 1% BSA, serum samples diluted 1:200 in PBS were added and incubated at 4°C overnight. The plates were then incubated with 1:10,000 goat anti-mouse IgG-HRP conjugate (Thermo Fisher Scientific, USA) for 1 h at 37°C. The color reaction was developed with 3,3′,5,5′-tetramethylbenzidine, TMB, (Sigma-Aldrich, USA), at OD 450 nm. Neutralizing antibodies to hMPV were measured by a plaque reduction neutralization assay, as previously described (38). The neutralizing antibody titers were defined as the reciprocal of the highest serum dilution at which ≥ 50% reduction in CPE was observed. The lowest detectable titer was 2.5 log_2_. Samples with non-detectable titers were assigned a value of 2 log_2_.

### Statistical analysis

The data were evaluated using ANOVA and two-tailed unpaired Student’s t-test for samples with unequal variances to determine significant difference between each set of two groups (GraphPad Prism 5.02; GraphPad Software, Inc., San Diego, CA). Results are expressed as mean ± standard error of the mean for each experimental group unless otherwise stated. p<0.05 value was selected to indicate significance. All experiments were repeated at least three times, data in figures are shown from a representative experiment with n = 3-6 mice/each group.

## Results

### Effect of G protein deletion on clinical disease, viral replication and lung pathology

**Figure 1:**
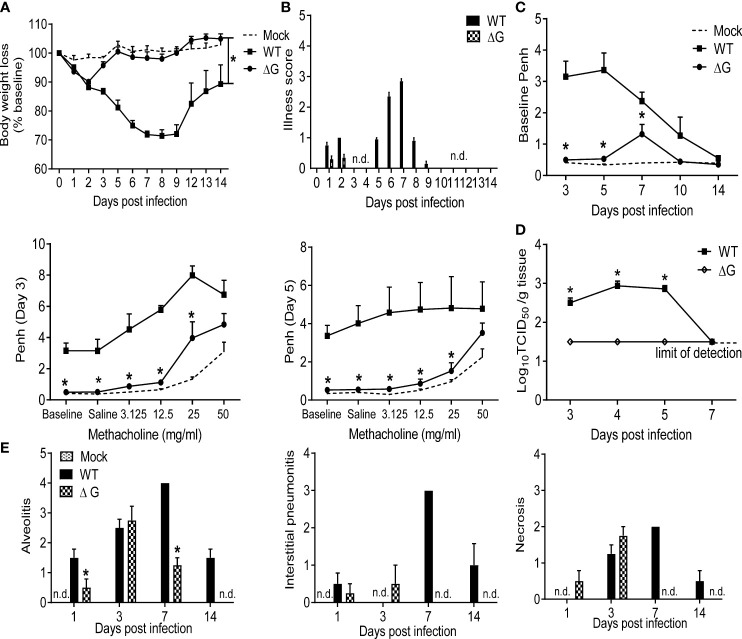
Clinical disease and virus replication. 6-8 wk old female BALB/c mice were either infected with rhMPV (-WT or -ΔG) or mock infected. **(A)** Change in body weight was measured over a period of 14 days. Body weight is expressed as percentage of baseline weight. **(B)** Illness score for infected mice infected was assessed daily by two observers using a grading scale from 0 to 5. **(C)** Baseline airway obstruction (left panel) and obstruction post-methacholine challenge (two right panels) was measured over a period of 14 days by unrestrained plethysmography. Day 3 and 5 p.i. are shown. **(D)** Infected mice were sacrificed at days 3, 4, 5 and 7 p.i. to determine viral titers by TCID_50_ assay. The lower limit of detection of this assay is 1.5 log10/gram of tissue. **(E)** Lungs were formalin-fixed for slide preparation, and hematoxylin and eosin stained. Alveolitis, interstitial pneumonitis and necrosis were scored on lung sections by a pathologist. Data are expressed as mean ± SEM of four to six animals/group and is representative of one of three independent experiments. *P<0.05 when comparing rhMPV -ΔG to -WT infected mice. rhMPV -ΔG =ΔG; rhMPV -WT = WT. n.d., not detected.

BALB/c mice were infected with rhMPV (-ΔG or -WT) or mock infected (PBS) and body weight loss was measured daily over the following 14 days. Infection of mice with WT virus showed the typical biphasic disease, with an initial phase of 2-3 days body weight loss followed by a second more severe phase of body weight loss (26). On the other hand, rhMPV -ΔG-infected mice demonstrated significantly reduced body weight loss with a complete absence of the second phase (p<0.05) (**Figure 1A** http://journals.plos.org/plosone/article?id=10.1371/journal.pone.0078849-pone-0078849-g001). Signs of illness, including ruffled fur, hunched appearance, and reduced movement, which closely parallels the body weight loss curve during hMPV infection, were prominent during infection with rhMPV -WT virus, lasting over a period of nine days, while minimal illness scores and body weight loss were observed only during the first two days p.i. in the rhMPV -ΔG-infected mice (**Figure 1B**). Infection with rhMPV-WT led to a significant increase in the baseline Penh values, indicating increased obstruction of the airways, compared to mock-infected mice, starting at day 3, peaking on day 5 and then gradually returning to baseline by day 14 p.i. In addition, rhMPV-WT infection was associated with significant dose-dependent increase in AHR in response to aerosolized methacholine (**Figure 1C**). Mice infected with rhMPV-ΔG did not manifest airway obstruction, with the exception of day 7 p.i. and had significantly reduced methacholine-induced AHR (**Figure 1C**). As shown in **Figure 1D**, rhMPV-WT replicated efficiently in mouse lungs, with a peak of viral titer at day 4 p.i., which declined to undetectable levels by day 7 p.i. On the other hand, the rhMPV- ΔG virus was restricted in replication, with viral titers below the lower limits of detection (<1.5 log10 TCID50/g tissue) at all time points tested. Histopathology analysis of H&E-stained lung sections showed that alveolitis was significantly greater at days 1, 7 and 14 p.i. in mice infected with rhMPV-WT compared to those infected with the ΔG mutant (**Figure 1E**). Similarly, there was a complete absence of interstitial pneumonia or cell necrosis at day 7 and 14 in rhMPV -ΔG-infected mice compared to those infected with rhMPV -WT virus.

### Modulation of cytokine, chemokine and interferon production by hMPV G protein

**Figure 2:**
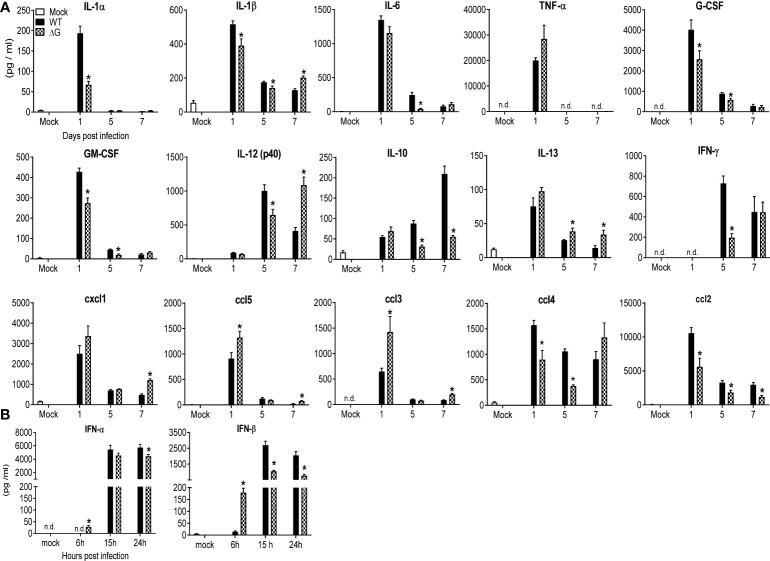
BAL fluid levels of pro-inflammatory cytokines, chemokines and type I IFNs. Mice were either infected with rhMPV-WT or -ΔG or mock infected and sacrificed at various time points p.i. to collect BAL fluid. **(A)** Levels of cytokines and chemokines in BAL fluid were measured by Bio-Plex. **(B)** Levels of IFN-α/β were measured by ELISA. Data are expressed as mean ± SEM of four to six animals/group and is representative of one of three independent experiments. *P<0.05 when comparing rhMPV- ΔG infected mice to rhMPV- WT infected mice. rhMPV -ΔG =ΔG; rhMPV -WT = WT. n.d., not detected.

To determine the role of hMPV G protein in modulating lung inflammation, BAL fluid from mock, rhMPV-WT and -ΔG infected mice was collected at day 1, 5 and 7 p.i. and assessed for cytokine and chemokine levels. Levels of several of the proinflammatory and immunoregulatory cytokines were significantly lower in rhMPV-ΔG infected mice, compared to rhMPV -WT (**Figure 2A**). These included IL-1α and -1β, granulocyte-macrophage colony-stimulating factor (GM-CSF), granulocyte-colony stimulating factor (G-CSF), IL-10, IL-12p40 and gamma interferon (IFN-γ). We observed a trend for early increased TNF-α but significant lower levels of IL-6 (day 5) in rhMPV-ΔG infected mice compared to WT-infected. At later time point of infection (day 7 p.i.) concentrations of IFN-γ and IL-12p40 in BAL of rhMPV-ΔG mice were similar or higher, respectively, compared to rhMPV -WT-infected ones. Interestingly, IL-13 levels were higher at all time point tested in ΔG-infected mice. Absence of G protein was associated with significantly higher chemokine levels in BAL cxcl1 (KC), ccl5 (RANTES) and ccl3 (MIP-1α), and lower levels of ccl2 (MCP1) and ccl4 (MIP-1β) (**Figure 2A**). Finally, we observed an early induction of type I interferons (IFN-α and -β) at a very early time point (6h p.i.) of rhMPV-ΔG infection, which was not sustained at later time points, as the levels of type I interferons, in particular IFN-β, were significantly greater in rhMPV -WT-infected mice compared to rhMPV-ΔG (**Figure 2B**).

### Effect of G protein deletion on lung immune cell frequency and activation and humoral antibody production

**Figure 3:**
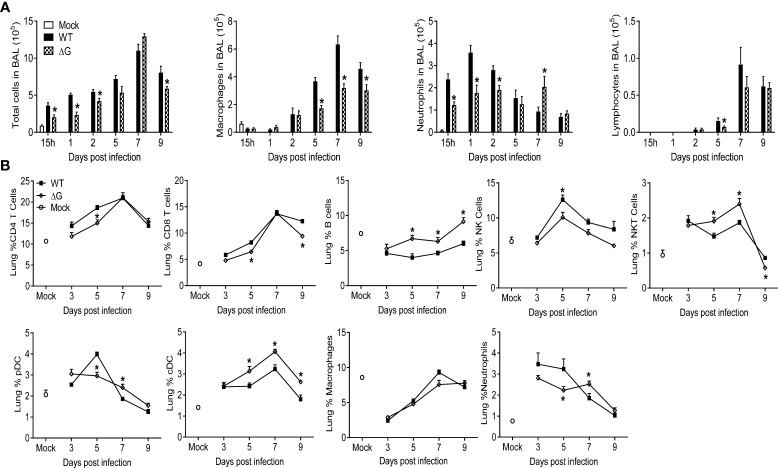
Cell recruitment in BAL and lung tissue. Mice were either infected with rhMPV-WT or rhMPV- ΔG/WT or mock-infected and sacrificed at various time points p.i. to collect BAL fluid and lungs. **(A)** BAL fluid was subjected to cytospin and analyzed for total and differential cell counts. **(B)** Cells were isolated from lungs and stained for cell-type specific CD markers and live cells analyzed by flow cytometry. Data are expressed as mean ± SEM of four mice/group and is representative of one of three independent experiments. * P<0.05 when comparing rhMPV- ΔG infected mice to rhMPV- WT infected mice. rhMPV -ΔG =ΔG; rhMPV -WT = WT.

To assess the cellular trafficking to the lungs in response to rhMPV-WT or -ΔG infection, BAL fluid and lung tissue were analyzed at several time points p.i. In the absence of G protein, the overall cellular recruitment in BAL fluid in response to infection was significantly lower at all the time points except days 5 and 7 p.i. (which represents the peak of inflammation during hMPV infection), when it was similar in both groups (**Figure 3A**). BAL fluid from mock- infected mice consisted mainly of macrophages, whereas infection with rhMPV-WT induced marked neutrophilia for the first three days p.i., followed by recruitment of monocytes/macrophages and lymphocytes at later time points of infection. The number of both neutrophils and macrophages in BAL were lower in rhMPV -ΔG-infected mice compared to rhMPV -WT, with the exception of neutrophils at day 7, which were higher in rhMPV -ΔG-infected mice (**Figure 3A**). Lymphocyte recruitment, which starts later during infection, was slightly lower in rhMPV -ΔG-infected mice at day 5 p.i., compared to WT, with similar levels at later time points of infection (**Figure 3A**).

To better define the immune cell response to rhMPV-WT and -ΔG infection, we performed flow cytometric analysis of lung T and B cells, natural killer (NK) cells, plasmacytoid dendritic cells (pDC) and conventional dendritic cells (cDC), as well as analysis of macrophages and neutrophils (**Figure 3B**). Compared to rhMPV -WT-infected mice, the frequency of both CD4^+^ and CD8^+^ T cells was lower in rhMPV -ΔG infected mice at early time points, returning to levels similar to WT infected mice by day 7 p.i. The percentage of NK cells was consistently higher in rhMPV -WT-infected mice at all time point analyzed. On the other hand, starting at day 5 p.i. the percent of B cells, NK and cDC cells was significantly higher in rhMPV -ΔG-infected animals compared to the rhMPV -WT-infected ones. Percent of pDC cells was similar in both groups except at day 5 p.i., when it was significantly reduced in the lung of rhMPV- ΔG infected mice. Similar to the findings in BAL fluid, mice infected with rhMPV- ΔG had smaller percent of neutrophils in the lung at earlier time points but a greater percent at day 7 p.i., compared to WT-infected mice.

**Figure 4:**
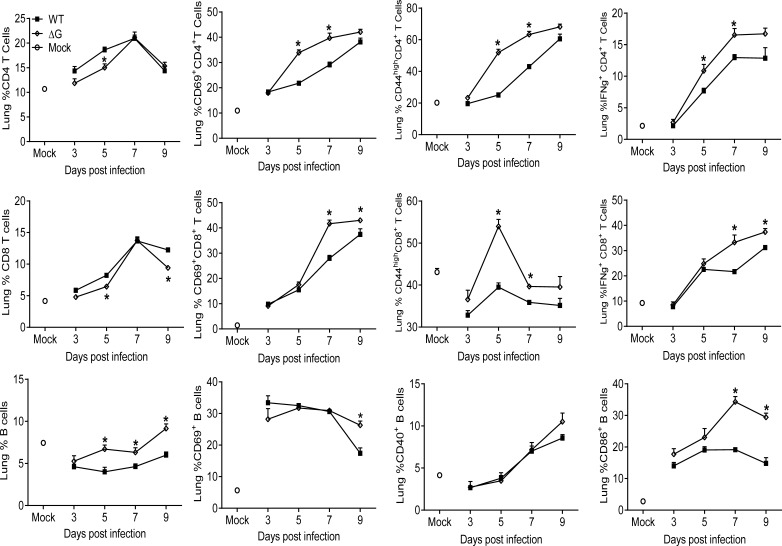
Lymphocyte recruitment and activation. Mice were either infected with rhMPV-WT or -ΔG or mock-infected and sacrificed at various time points p.i. to collect lungs. Single cell suspension of lungs was obtained and stained for CD4 (CD3^+^CD4^+^) and CD8 (CD3^+^CD8^+^) T cells and B cells (CD19^+^B220^+^). Lymphocytes were further stained for expression of various activation markers (CD69, CD44, CD40, CD86). IFN-γ produced by T cells was detected by intracellular staining upon stimulation with PMA/ionomycin and analyzed by flow cytometry. * P<0.05 when comparing rhMPV- ΔG infected mice to rhMPV- WT infected mice. rhMPV -ΔG =ΔG; rhMPV -WT = WT.

Although the frequency of T cells in the lung was lower in rhMPV- ΔG -infected mice compared to WT, both CD4^+^ and CD8^+^ T cells from the ΔG group expressed significantly higher levels of the activation markers CD69 and CD44 compared to lung T cells from rhMPV -WT-infected mice (**Figure 4**). In addition, ΔG CD4^+^ and CD8^+^ T cells had increased intracellular levels of IFN-γ on days 5, 7 and 9 and 7 and 9, respectively, compared to WT T cells. The marked increase in B cell recruitment observed in response rhMPV-ΔG infection was paralleled by an upregulation of the activation markers CD69 on day 9 p.i. and CD86 on days 7 and 9 p.i., compared to rhMPV -WT infection (**Figure 4**).

**Figure 5:**
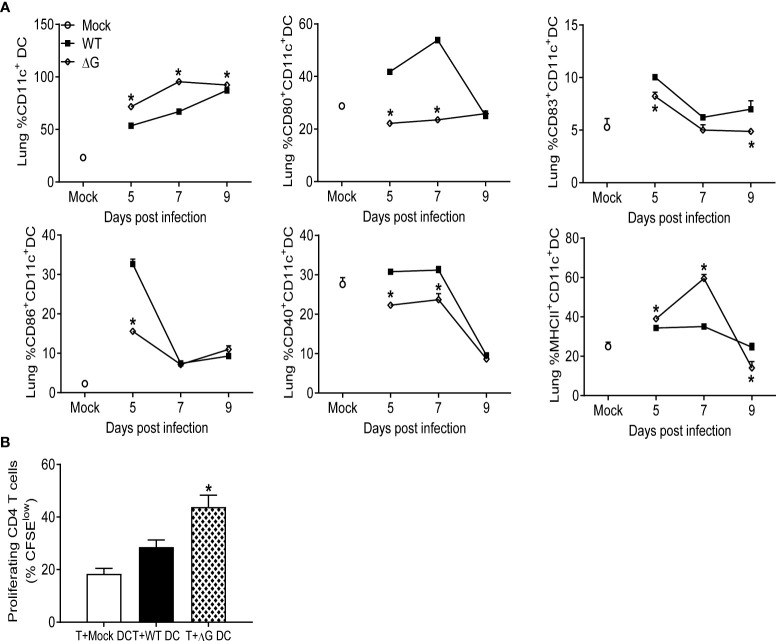
Dendritic cells recruitment to the lung, activation, and proliferation. Mice were either infected with rhMPV-WT or -ΔG or mock-infected and sacrificed at various time points p.i. to collect lungs. Single cell suspension of lungs was obtained and stained for DC markers. **(A)** CD11c^+^DCs population was enriched from the lung cells and analyzed by flow cytometry for the expression of activation markers (CD80, CD83, CD86, CD40, MHC class II). **(B)** CD11c^+^ cells isolated from lungs of infected mice at day 7 p.i. were loaded with 10 µg/mL of OVA peptide for 2h prior to co-culture with T cells. CD4+ T cells isolated from spleen of DO11.10 mice were labeled with CFSE and co-cultured with DCs at a ratio of 1∶2 (DC:T). T cell proliferation was measured by CFSE dilution. Cultures without antigen served as controls. The bar graph shows the percentage of proliferating (CFSE low) T cells among the total CD4+ T cell population. * P<0.05 when comparing rhMPV- ΔG infected mice to rhMPV- WT infected mice. rhMPV -ΔG =ΔG; rhMPV -WT = WT.

As we found that cDCs were recruited in significantly higher numbers in the lungs of rhMPV-ΔG infected animals compared to rhMPV -WT-infected, we also assessed their activation/co-stimulatory status and function. Lung CD11c^+^ positive cells were isolated from infected mice by magnetic sorting and flow cytometry was used to assess the expression of costimulatory/activation markers, as well as their ability to induce T-cell proliferation *in vitro*. At days 5 and 7 p.i. the percentages of sorted CD11c^+^ and their expression of MHC-II were significantly higher in rhMPV- ΔG infected mouse lungs compared to the rhMPV- WT -infected ones (**Figure 5A**). However, these CD11c^+^ cells isolated from ΔG-infected lungs expressed lower levels of the all the tested co-stimulatory molecules, including CD40, CD80, CD83 and CD86, was markedly lower in rhMPV -ΔG compared to -WT-infected lungs. To investigate antigen presenting capacity, CD11c^+^DCs isolated from mock, rhMPV- WT- and ΔG-infected mice on day 7 p.i., were loaded with OVA peptide and co-cultured with purified and CFSE labeled splenic CD4^+^ T cells from DO11.10 mice. Incubation of lung CD11c^+^DCs from mock and rhMPV-infected mice with DO11.10 CD4^+^ T cells resulted in a robust T cell proliferation, as indicated by the increase in percent of CFSE low cells, with somehow surprisingly higher proliferation observed in cultures of CD11c^+^DCs isolated from rhMPV- ΔG infected mice compared to WT (**Figure 5B**).

### Antibody production and protection against virus challenge following rhMPV-ΔG and -WT infection

**Figure 6:**
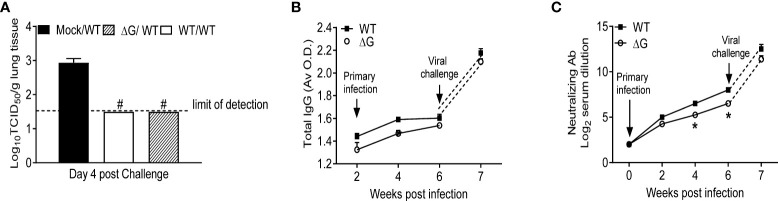
Virus replication and antibody titer in response to virus challenge. Mock and previously rhMPV-WT or -ΔG infected mice were challenged with rhMPV-WT virus 6 weeks after primary infection. **(A)** Mice were sacrificed on day 4 post challenge (peak of viral replication), and lungs were harvested for determining viral titers by TCID_50_ assay. **(B)** Total hMPV-specific IgG titers in serum were determined by ELISA. **(C)** hMPV-specific neutralizing antibody titer was determined by plaque neutralization assay. Data are expressed as mean ± SEM of four animals/group and is representative of one of two independent experiments. *P<0.05 when comparing rhMPV-WT and -ΔG; ^#^P<0.05 when comparing rhMPV-WT and -ΔG and mock- mice challenged with rhMPV -WT. rhMPV -ΔG =ΔG; rhMPV -WT = WT; Mock/rhMPV -WT = Mock/W; rhMPV -ΔG/rhMPV –WT =ΔG/WT; rhMPV -WT/rhMPV -WT = WT/WT.

**Figure 7:**
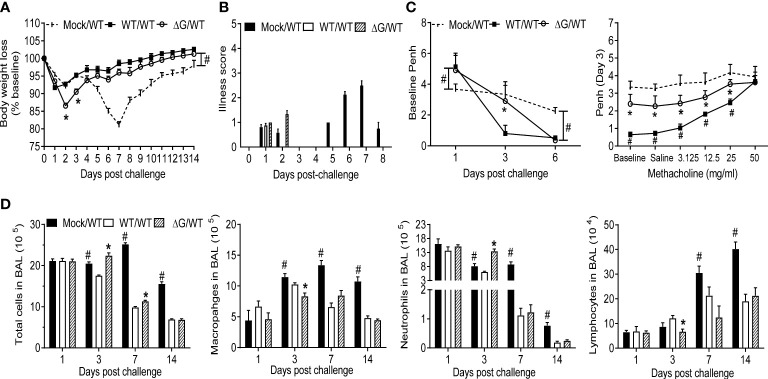
Clinical disease, cellular immune responses in response to virus challenge. Mock and with rhMPV-WT or -ΔG infected mice were challenged with rhMPV -WT virus 6 weeks after primary infection and sacrificed at various time points p.i. to collect BAL fluid. Change in body weight **(A)** and illness score **(B)** were measured over a period of 14 days. Data are expressed as mean ± SEM of four animals/group and the figure represents cumulative data from two independent experiments. **(C)** Penh values were measured at baseline (airway obstruction) and post-methacholine challenge by unrestrained plethysmography. Data are expressed as mean ± SEM of four animals/group and is representative of one of two independent experiments. **(D)** BAL fluid was subjected to cytospin and analyzed for total and differential cell counts. *P<0.05 when comparing rhMPV -WT and -ΔG mice challenged with rhMPV -WT.; ^#^P<0.05 when comparing rhMPV (-WT and -ΔG) challenged with rhMPV -WT and mock- mice challenged with rhMPV -WT. Mock/rhMPV -WT = Mock/WT; rhMPV -ΔG/rhMPV –WT =ΔG/WT; rhMPV -WT/rhMPV -WT = WT/WT.

To investigate protection against reinfection, BALB/c mice previously mock-infected or infected with either rhMPV-WT or -ΔG were challenged with rhMPV -WT virus six weeks after primary infection. Compared to mock/rhMPV -WT mice both rhMPV -WT/-WT and rhMPV -ΔG/-WT mice rapidly cleared infection as lung viral titers were below the lower limit of detection at the peak of viral replication, i.e. day 4 p.i. (**Figure 6A**). In both the rhMPV -WT/-WT and rhMPV -ΔG/-WT groups there were comparable levels of hMPV-specific total IgG (**Figure 6B**) and neutralizing antibody (**Figure 6C**) within a week post challenge, which were significantly higher than the levels detected 6 weeks after primary infection. Of interest, the level of neutralizing antibody induced by rhMPV-ΔG during primary infection was somewhat less at week 4 and 6 p.i., compared to levels induced by rhMPV-WT. After viral challenge, all the groups demonstrated an initial phase of body weight loss around day 2 p.i., which was more pronounced in the rhMPV- ΔG/WT group (**Figure 7A**). On day 5 post challenge, only mice previously mock-infected (mock/rhMPV -WT) demonstrated a second phase of body weight loss, reaching peak body weight loss by day 7 p.i., as expected in response to a primary infection (**Figure 7A**). Signs of illness were visible and similar in all the groups on day 1 post challenge, with a slight increase in the rhMPV- ΔG/WT group on day 2 (**Figure 7B**). The illness score was zero for both rhMPV- WT/-WT and rhMPV- ΔG/-WT infection groups, compared to mock/rhMPV -WT, during the following days of the virus challenge (**Figure 7B**). In parallel with the observed initial phase of body weight loss, all the groups developed baseline airway obstruction (i.e. without methacholine challenge) on day 1, which was significantly higher in the/WT rhMPV- WT/WT and/WT rhMPV- ΔG/WT groups compared to the mock/rhMPV -WT group (**Figure 7C**). Airway obstruction decreased in the rhMPV -WT/-WT group by day 3, whereas it continued to be significantly higher in the rhMPV- ΔG/WT and mock/rhMPV -WT groups. By day 6 Penh had returned to baseline level in the rhMPV- WT/-WT and ΔG rhMPV- ΔG/-WT groups, while some degree of airway obstruction was still present in the mock/WT group (as expected by our results in primary infection, **Figure 1C**). Consistent with the data for baseline airway obstruction, at day 3 post-challenge mice in the mock/rhMPV -WT group had higher AHR compared to the other two groups in response to methacholine challenge, with animals in the ΔG/WT group showing higher AHR compared to the rhMPV- WT/-WT group.

### BAL cytokines and cells following viral challenge

**Figure 8:**
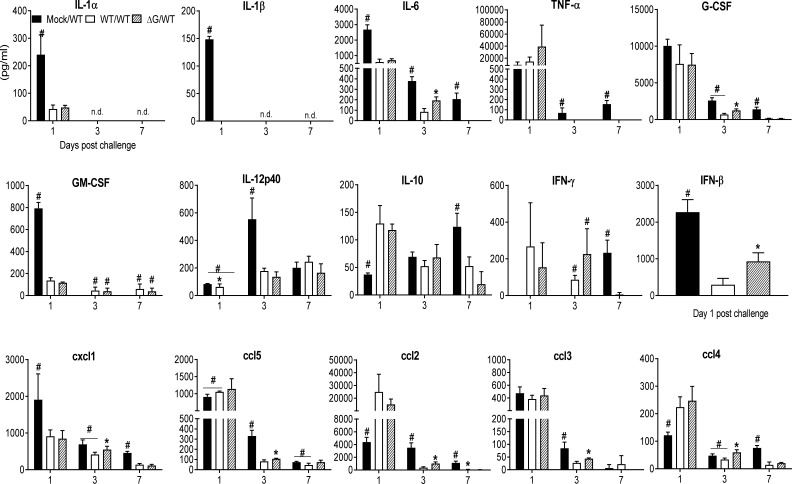
BAL fluid levels of pro-inflammatory cytokines, chemokines and type I IFNs in response to virus challenge. Mock and previously rhMPV-WT or -ΔG infected mice were challenged with rhMPV-WT virus as previously described in Material and Methods. Levels of cytokines and chemokines in BAL fluid were measured by Bio-Plex. Levels of IFN-β were measured by ELISA. Data are expressed as mean ± SEM of four to six animals/group and is representative of one of three independent experiments. *P<0.05 when comparing rhMPV -WT and -ΔG mice challenged with rhMPV -WT.; ^#^P<0.05 when comparing rhMPV (-WT and -ΔG) challenged with rhMPV -WT and mock- mice challenged with rhMPV -WT. Mock/rhMPV -WT = Mock/WT; rhMPV -ΔG/rhMPV –WT =ΔG/WT; rhMPV -WT/rhMPV -WT = WT/WT. n.d., not detected.

BAL fluid was collected at several time points post challenge in the above-described groups of mice for quantitative analysis of cellular composition and cytokine concentration. Total number of BAL cells was similar in all the groups on day 1 post challenge, with the rhMPV -ΔG/-WT group having a significantly higher cellular recruitment on day 3 compared to the other groups. By day 7 and 14, BAL cellular recruitment in the rhMPV -WT/-WT and ΔG/WT groups was significantly reduced compared to the mock group (**Figure 7D**). Significant neutrophilia, with lower levels of macrophages and lymphocytes, was observed in the rhMPV- ΔG/-WT challenged group on day 3, compared to all the other groups. By day 7 and 14 post challenge, all cell types in the BAL were present in lower numbers in the rhMPV- WT/WT and rhMPV- ΔG/-WT groups, compared to the mock/WT group (**Figure 7D**). The overall proinflammatory cytokine response (IL-1α, IL-1β, IL-6), as well as GM-CSF levels, were significantly lower in the rhMPV- WT/-WT and rhMPV- ΔG/-WT groups compared to mock/WT on day 1 post challenge, with TNF-α levels being similar in all the groups (**Figure 8**). The regulatory cytokine IL-12p40 level was also lower in the rhMPV- WT/-WT and rhMPV- ΔG/-WT groups on day 3 post challenge compared to the mock/rhMPV -WT group. On the other hand, concentrations of IL-10 on day 1 and IFN-γ on both day 1 and 3 post-challenge were significantly greater rhMPV- WT/-WT and rhMPV- ΔG/-WT groups compared to mock/WT (**Figure 8**). IFN-β levels were significantly higher in the rhMPV- ΔG/-WT group compared to the rhMPV- WT/-WT group, although significantly lower in both groups compared to the mock/WT group. As far as the concentrations of chemokines, cxcl5 (RANTES), ccl4(MIP-1β), and more strikingly ccl2 (MCP-1) were significantly increased, while cxcl1 (KC) was reduced in rhMPV- WT/-WT and rhMPV- ΔG/-WT groups compared to mock/WT group at day 1 post-challenge. Chemokine levels were usually lower in the rhMPV -WT and - ΔG groups compared to mock at subsequent days post challenge (**Figure 8**).

## Discussion

Using a mouse model of infection, we show that our recombinant hMPV with G protein deletion is strongly attenuated *in vivo*, as its replication in the lung was at the lowest levels of detection by TCID_50_ assays, and did not induce significant clinical disease and airway disfunction, compared to a recombinant hMPV wild type strain (rhMPV-WT). Similar attenuation of ΔG mutants has been previously observed in studies of the upper and lower respiratory tracts of hamsters and African green monkeys (18, 19, 39). Thus, our data confirms that even though G protein is not essential for viral entry and replication *in vitro*, it plays an important role in replication of hMPV and other pneumoviruses (RSV) in the airways of experimental animal models (19, 40–43).

The attenuated phenotype of our hMPV ΔG could be in part responsible for the reduced production of some of the inflammatory cytokines measured in mouse BAL fluid (**Figure 2**). For example, at the earliest time point tested (day 1) IL-1, G-CSF, GM-CSF were significantly reduced in ΔG-infected mice. However, this was not the case for TNF or IL-6. We have shown previously that TNF of IL-6 are mainly produced by alveolar macrophages in hMPV infection (44), thus suggesting that the initial interaction of hMPV with these cells may be intact even in absence of the G protein. Similarly, this could also explain the fact that at the early time point (day 1) the inducible chemokines cxcl1, ccl5 and ccl3 were produced at comparable and even higher levels by the ΔG virus. Except for ccl2, which was consistently lower at all time point tested in rhMPV- ΔG virus-infected mice, all other chemokines were detected at higher concentrations at day 7 post-inoculation in rhMPV -ΔG virus infection. The exact cellular source(s) of many chemokines measured in the BAL of infected mice is not known, as other cells such airway epithelial cells and immune cells may contribute to the early release of ccl4 and ccl2, both of which were reduced in absence of G protein, or to the later increased production in absence of G protein, respectively. For example, neutrophils, which were significantly decreased at the beginning of infection and then increased in number in rhMPV- ΔG -infected mice at day 7 p.i., could represent an important source of the chemokines that were significantly changed (lower at the beginning and elevated later) in ΔG-infected mice (45).

IFN type I, which like TNF and IL-6 is significantly produced by alveolar macrophages in mice infected with hMPV (44), was detected at the earliest time point after inoculation (6h) only in absence of G protein, although it was subsequently reduced at 15 and 24h p.i. compared to WT virus, likely due to restriction of replication in airway epithelial cells, which also account for a significant proportion of type I IFN present in the BAL of hMPV-infected mice (44), although we did not attempt to determine viral titers at these early time points due to the limitations of TCID_50_ assay at the beginning of infection (detection of inoculated virus). Cytokines associated with the adaptive immune response were also affected by lack of G protein. In particular, we found that at the peak of disease, IL-10 levels were lower and IL-12p40 higher in the BAL of mice infected with rhMPV-ΔG compared to rhMPV -WT (**Figure 2**). The role of IL-10 in hMPV infection is unknown, but in infections associated with RSV, a virus that cause similar respiratory disease in children, IL-10 has been shown to play a clear role in suppressing RSV-induced immunopathology, and CD4 T cells in different experimental models are major producer of this cytokine (46). IL-12p40 is produced primarily by activated macrophages, dendritic cells, and neutrophils. IL-12p40 also plays a pivotal agonistic role in initiating the immune response and performs several immunostimulatory functions including dendritic cell migration, macrophage-mediated inflammation, and to trigger IFN-γ production by NK and T cells (47). In mouse models of hMPV infection IL-12p40 appears to play an important function in limiting viral-induced disease, reducing lung inflammation, airway mucus production and regulating pulmonary cell infiltration and cytokine release (48).

In our study, the frequency of both lung CD4^+^ and CD8^+^ T cells expressing markers of cell activation (likely representing virus-specific T cells) was higher in rhMPV -ΔG-infected mice compared to rhMPV -WT-infected mice (**Figure 4**). The percent of IFN-γ positive CD4^+^ and CD8^+^ T cells was also greater in ΔG infection. Whether these activated T cell responses contribute to a more robust antiviral response in rhMPV- ΔG -infected mice remains to be determined. Previous studies have shown both CD4^+^ and CD8^+^ T cells cooperate synergistically in hMPV eradication during primary infection, with the CD4^+^ T cells contributing more to clinical disease and lung pathology and CD8^+^ T cells contributing significantly to viral clearance (26). Hence, the enhanced activation state of the T cells and generation of high frequency of activated memory T cells, could ensure a more rapid clearance of the virus in rhMPV- ΔG -infected mice. Other lung immune cells were found at higher frequency in ΔG infection, specifically NKT cells as early as day 5 post inoculation. NKT cells play a major function in communication between innate and adaptive immunity, by enhancing DC and B cell functions and ensuring optimal virus-specific T and B cell responses in the mucosal and systemic compartments (49–51). Concomitantly we noticed increased percent of DCs in the lung in response to infection with rhMPV -ΔG in comparison to the rhMPV- (**Figure 3**). ΔG DCs appeared to express higher levels of MHC-II (**Figure 5A**), and to induce stronger T cell proliferation *ex vivo*, compared to those isolated from rhMPV -WT-infected mice (**Figure 5B**), suggesting that G protein may be capable to inhibit DC-mediated activation of T cell responses during infection. In another study with human monocyte derived dendritic cells (MDDCs), infection *in vitro* with hMPV lacking both G and SH proteins, did not seem to influence the MDDCs activation, but induced a significantly stronger autologous CD4^+^ T cell proliferation compared to infection of MDDCs with hMPV WT virus (22, 52). This effect was shown to be mediated by inhibition of virus micropinocytosis in DC by SH and G glycoproteins, resulting in a reduced ability of the hMPV-stimulated DC to activate CD4+ T cells. We have previously shown that hMPV infection of human monocyte derived DCs decreases their capacity to stimulate T cell proliferation, albeit less efficiently compared to RSV (53). Lung B cells from ΔG-infected mice were also more frequent and significantly activated compared to WT-infected mice, as shown by the upregulation of CD86 marker, which might contribute to B lymphocyte co-stimulatory APC function. These activated B lymphocytes could induce antigen specific T cell proliferation and expression of activation markers thereby activating T cells (54–56).

Interestingly, we observed that the lack of G protein was associated with a significant lower number of neutrophils in BAL samples collected in the first two days of infection (**Figure 3A**), as reported in another study (57). We also observed a lower frequency of neutrophils in the lung of rhMPV -ΔG-infected mice, up to day 5 p.i. However, as we previously mentioned, BAL and lung neutrophils were found in slightly higher percent in ΔG-infected mice at day 7 (**Figure 3B**), possibly due to the increased production of chemokines including cxcl1 that was observed at that time point (**Figure 2**). These data suggest that neutrophils contribute to the manifestations of disease, lung inflammation and airway obstruction during the first couple of days of mice infection with hMPV and that G protein is an important virulence factor that affects neutrophil migration to the BAL and lung tissue.

As shown in **Figure 6**, the ΔG strain could induce the production of hMPV-specific IgG and neutralizing antibodies in mouse serum, and, although levels of neutralizing antibodies were a bit lower than those induced by the WT virus, similar to what previously reported in hamsters and non-human primates infected with rhMPV –ΔG (18, 19), the antibody response was comparably efficient in protecting mice from a challenge infection with hMPV. Indeed, mice challenged with rhMPV -WT 6 weeks after primary infection with rhMPV -WT or rhMPV -ΔG showed comparable boosted antibody response (albeit higher in the WT/WT group) and undetectable virus by in the lung by TCID_50_. In addition, the results of our studies in the challenge model suggest that prior infection with either rhMPV -WT or rhMPV- ΔG protected mice from hMPV reinfection in terms of body weight loss, airway disease, BAL cell inflammation, and cytokines release, with some minor differences between rhMPV -WT and rhMPV -ΔG. Specifically, the rhMPV -ΔG/-WT group showed a more pronounced body weight loss at early days after challenge, airway obstruction and increased AHR compared to the rhMPV -WT/-WT group. The reason for these differences is unclear at the moment, but we speculate that the rhMPV -ΔG/-WT may have some level of viral replication in the lung at earliest time points after challenge, which cannot be detected by the TCID_50_ assay.

In summary, results of this detailed study using a deleted hMPV mutant (ΔG) in a mouse model of infection support the evidence that the G glycoprotein of hMPV is an important virulence factor involved in the pathogenesis of lung disease, including airway obstruction and AHR. As shown by the analysis of BAL and lung cells, as well as cytokine production G protein is capable of affecting aspects of both innate and adaptive immunity. Despite highly attenuation characterized by low levels of replication in the lung, as shown in other animal models (15, 18, 19, 39), ΔG strain was able to induce neutralizing antibodies and like the fully competent WT strain to protect mice from a secondary hMPV challenge. However, with the limitations of a rodent model, hMPV challenged mice that had received ΔG as primary infection showed some signs of enhanced lung disease at the earliest time points, which were less evident in mice that had received the WT strain as primary infection. This finding should be further explored as vaccine platforms based on recombinants strains lacking G protein have been under consideration for hMPV vaccines.

## Data Availability Statement

The raw data supporting the conclusions of this article will be made available by the authors, without undue reservation.

## Ethics Statement

All procedures involving mice in this study were performed in accordance with the recommendations in the Guide for the Care and Use of Laboratory Animals of the National Institutes of Health. The protocol was approved by the Institutional Animal Care and Use Committee of the University of Texas Medical Branch at Galveston. The study has been approved by the UTMB IACUC (protocol number 0808049).

## Author Contributions

TV and TI contributed to the design of the experiments, performed the experiments and wrote the manuscript; AC and RG provided the overall conceptual design of these studies, reviewed and edited the final version of the manuscript. All authors contributed to the article and approved the submitted version.

## Funding

This work was partially supported by NIH grant AI079246. TV was supported by the James W. McLaughlin Fellowship Fund.

## Acknowledgments

We thank Tianshuang Liu for her technical support, and Cynthia Tribble for her help with manuscript editing and submission.

## Conflict of Interest

The authors declare that the research was conducted in the absence of any commercial or financial relationships that could be construed as a potential conflict of interest.

## Publisher’s Note

All claims expressed in this article are solely those of the authors and do not necessarily represent those of their affiliated organizations, or those of the publisher, the editors and the reviewers. Any product that may be evaluated in this article, or claim that may be made by its manufacturer, is not guaranteed or endorsed by the publisher.
